# RGD Peptide-Functionalized CMC Hydrogel Enhances Skin Wound Repair in Mice

**DOI:** 10.3390/molecules31203761

**Published:** 2026-10-19

**Authors:** Chen Liang, Shuyue Wang, Jeevithan Elango, Qing Yang, Bailin Li, Wenhui Wu, Xiaozhen Diao

**Affiliations:** 1 Department of Marine Pharmacology, College of Food Science and Technology, Shanghai Ocean University, Shanghai 201306, China; 2 Department of Biomaterials Engineering, Faculty of Health Sciences, UCAM—Universidad Católica San Antonio de Murcia, Campus de los Jerónimos 135, Guadalupe, 30107 Murcia, Spain; 3 Center of Molecular Medicine and Diagnostics (COMManD), Department of Biochemistry, Saveetha Dental College and Hospitals, Saveetha Institute of Medical and Technical Sciences, Saveetha University, Chennai 600077, India; 4 Shanghai Knowhub Technology Co., Ltd., Pudong New Area, Shanghai 201210, China; 5 Putuo Sub-Center of International Joint Research Center for Marine Biological Sciences, Zhoushan 316104, China; 6 Marine Biomedical Science and Technology Innovation Platform of Lin-Gang Special Area, Shanghai 201306, China

**Keywords:** carboxymethyl chitosan composite hydrogel, RGD peptide, hyaluronic acid, antibacterial activity, hemostasis, inflammatory regulation, wound repair

## Abstract

Disruption of the wound microenvironment by bacterial infection and excessive inflammation severely delays tissue repair, making multifunctional hydrogels that combine infection control and immune regulation highly desirable. Building on our previously reported carboxymethyl chitosan (CMC) hydrogel incorporating hyaluronic acid (HA) and RGD peptide (RGD), this study extended its evaluation to antibacterial activity, hemostatic behavior, and in vivo wound repair. Four formulations—CMC, CMC-HA, CMC-RGD, and CMC-HA-RGD—contained 1 wt% CMC with 0.9 wt% HA and/or 0.02 mg/mL RGD. Antibacterial activity against *Escherichia coli* (*E. coli*) and *Staphylococcus aureus* (*S. aureus*) was assessed by agar diffusion, while hemostatic behavior and wound repair were evaluated using mouse tail-transection and aseptically established full-thickness skin-wound models. Histology, immunohistochemistry, and serum enzyme-linked immunosorbent assays were used to assess tissue repair and inflammatory markers. CMC-HA-RGD produced inhibition-zone areas of 2.0 cm^2^ against *E. coli* and 6.0 cm^2^ against *S. aureus*. The RGD-containing formulations showed reductions in visible bleeding. Wound closure with CMC-HA-RGD reached 41.39%, 71.18%, and 95.56% on days 3, 7, and 14, respectively, compared with 16.47%, 58.18%, and 79.85% for CMC. Histology showed more complete epidermal–dermal architecture and denser collagen deposition. On day 14, relative to CMC, CMC-HA-RGD showed 35.37% and 37.74% lower interleukin-1β (IL-1β)- and tumor necrosis factor-α (TNF-α)-positive areas, respectively, while the collagen I-positive area increased from 63.36% to 72.58%. Serum interferon-γ (IFN-γ), IL-1β, and TNF-α concentrations were 9.08%, 18.01%, and 11.85% lower than those in the CMC group. Collectively, incorporating HA and RGD improved the antibacterial and hemostatic performance of the CMC hydrogel, attenuated local and systemic inflammatory responses, and promoted tissue remodeling, supporting its potential as a multifunctional wound dressing.

## 1. Introduction

Skin wounds represent a major clinical challenge due to their complex healing process and susceptibility to microbial infection [1,2]. Normal wound healing is a highly coordinated biological process involving overlapping phases, such as hemostasis, inflammation, proliferation, and tissue remodeling [3,4]. Immediately after injury, inflammatory responses are initiated to remove damaged tissues and invading microorganisms, followed by granulation tissue formation, angiogenesis, extracellular matrix (ECM) deposition, and re-epithelialization [3,5]. However, excessive or prolonged inflammation caused by bacterial contamination can disturb this physiological sequence, resulting in delayed wound closure, impaired tissue regeneration, and even chronic wound formation [6,7,8]. Therefore, maintaining an appropriate balance between antibacterial defense and immune regulation is essential for achieving effective wound repair [2,6].

Bacterial infection is one of the primary factors responsible for impaired wound healing [9,10]. The disruption of the skin barrier after injury provides favorable conditions for bacterial adhesion and proliferation [11]. Among various pathogenic microorganisms, *Staphylococcus aureus* (*S. aureus*) and *Escherichia coli* (*E. coli*) are two of the most frequently isolated bacteria from infected wounds [8,12]. *S. aureus*, a typical Gram-positive bacterium, can form biofilms and release virulence factors that interfere with immune responses and tissue regeneration [13,14]. In contrast, *E. coli*, a representative Gram-negative bacterium, can induce inflammatory activation through bacterial components such as lipopolysaccharides, resulting in excessive production of inflammatory cytokines [15,16]. Persistent bacterial colonization not only causes direct tissue damage but also prolongs inflammatory responses, ultimately preventing the transition from the inflammatory phase to the proliferative phase of wound healing [17]. Thus, the development of wound dressings with effective antibacterial properties is highly desirable [18,19,20].

Hydrogels have attracted considerable attention as wound dressing materials due to their high water content, three-dimensional network structure, and ability to provide a moist environment favorable for tissue regeneration [21,22]. However, conventional hydrogel systems mainly function as physical barriers and often exhibit limited antibacterial activity, making them insufficient for preventing bacterial infection during wound healing [18]. To overcome this limitation, various antibacterial agents have been incorporated into hydrogel systems, including antibiotics, metal ions, and nanoparticles [23,24]. Although these strategies can effectively inhibit bacterial growth, concerns regarding antibiotic resistance, cytotoxicity, and long-term biosafety remain [25,26,27]. Consequently, naturally derived biomaterials with intrinsic antibacterial properties and immunomodulatory functions have become promising candidates for next-generation wound dressings.

Carboxymethyl chitosan (CMC), a water-soluble derivative of chitosan, has been widely investigated in biomedical applications due to its excellent biocompatibility, biodegradability, and inherent antibacterial properties [28,29,30,31]. The positively charged amino groups within CMC can interact with negatively charged bacterial membranes, resulting in membrane destabilization and inhibition of bacterial proliferation [32,33]. In addition, CMC possesses favorable water retention ability and excellent processability, making it an attractive candidate for constructing hydrogel-based wound dressing systems [34,35,36]. Hyaluronic acid (HA), an important component of the extracellular matrix, plays essential roles in maintaining tissue homeostasis, regulating inflammatory responses, and promoting tissue remodeling [37,38,39]. HA can provide a hydrated microenvironment for cell migration and proliferation, while also participating in the regulation of immune responses during wound healing [40,41,42]. RGD peptides (RGD), as extracellular matrix-derived adhesion motifs, can enhance cell–matrix interactions by binding to integrin receptors, thereby improving the biological activity of biomaterials [43,44,45]. In our previous study, we developed a biofunctional CMC hydrogel incorporating HA and RGD peptides and characterized its physicochemical properties and cellular responses relevant to wound repair [46]. The present study does not repeat the physicochemical characterization of this previously established hydrogel system; rather, the previously reported structural characterization is cited only to define the material system and provide the necessary structural context. However, its antibacterial activity, hemostatic performance, and functional efficacy in in vivo wound repair had not been systematically evaluated. In particular, its effects on tissue regeneration, collagen deposition, and inflammatory-marker responses during wound healing remained to be further investigated. Accordingly, the present work extends the evaluation of this hydrogel to antibacterial, hemostatic, and in vivo wound-repair models, and all antibacterial, hemostatic, histological, immunohistochemical, cytokine, and wound-healing data reported herein were newly generated in the present study. This expanded evaluation provides a more comprehensive assessment of the potential of the CMC-HA-RGD hydrogel as a multifunctional wound dressing material.

Based on the above hypothesis, this study, the present study extended the functional and biological evaluation of the previously developed CMC-HA-RGD hydrogel. The antibacterial activity of CMC, CMC-HA, CMC-RGD, and CMC-HA-RGD hydrogels was evaluated against *E. coli* and *S. aureus*. A mouse tail-transection model was used to assess hemostatic behavior, and an aseptically established full-thickness skin-wound model was employed to evaluate wound closure and tissue repair. Histological analysis, immunohistochemistry, and serum cytokine measurements were further performed to assess tissue regeneration, collagen deposition, and changes in selected local and systemic inflammatory markers. Thus, the present study expands the scope of our previous work from physicochemical and cellular characterization to antibacterial, hemostatic, and in vivo wound-repair evaluation, providing a more comprehensive assessment of the functional performance of the CMC-HA-RGD hydrogel for skin wound repair.

## 2. Results

### 2.1. Hydrogel Formulations and Structural Context

The CMC-based hydrogel formulations evaluated in the present study were prepared using the hydrogel system established in our previous work. No new physicochemical characterization data are presented in this section. Instead, the previously reported formulation and structural information are summarized briefly to define the hydrogel system used in the subsequent functional and biological experiments. All four formulations contained CMC as the primary polymeric matrix, with HA and/or RGD incorporated during EDC/NHS-assisted gelation, resulting in the CMC, CMC-HA, CMC-RGD, and CMC-HA-RGD formulations. The physicochemical characterization of these formulations, including morphological analysis, zeta-potential measurement, thermal analysis, FTIR characterization, and in vitro cellular evaluation, was previously reported in our earlier study [46].

**Figure 1:**
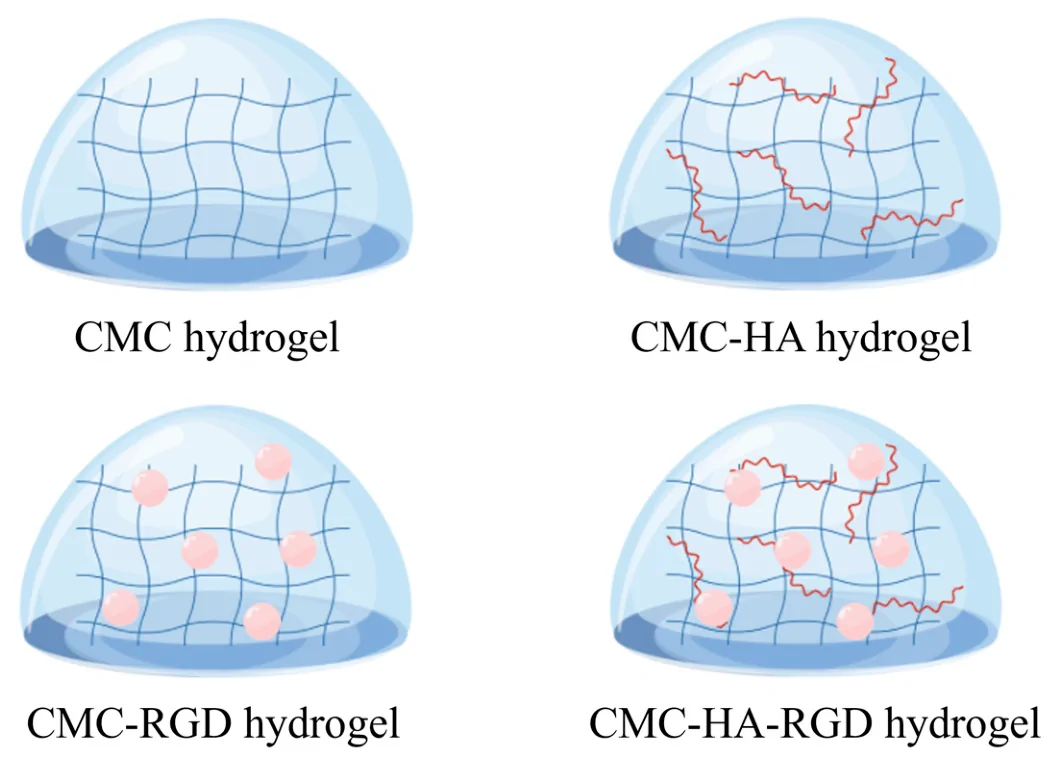
Structural pattern diagrams of CMC-based hydrogels.

Based on the previously established preparation process and structural characterization, the hydrogel network was considered to involve EDC/NHS-assisted covalent interactions together with non-covalent interactions, including hydrogen bonding and electrostatic interactions. Incorporation of HA and/or RGD introduced additional functional groups and intermolecular interactions into the CMC-based network, as schematically illustrated in Figure 1.

The FTIR information summarized here is derived from our previous publication [46] and is included solely to provide structural context for the material system rather than as newly acquired characterization data. In the previous study, FTIR analysis showed formulation-dependent spectral changes in the approximately 1600–1621 cm^−1^ region after incorporation of HA and/or RGD. These changes were associated with alterations in the chemical environment of carbonyl- and carboxylate-containing groups and, together with the established preparation process, supported the presence of interactions within the hydrogel network.

In contrast to the previously published physicochemical characterization, all functional and biological results presented in the following sections—including antibacterial activity, hemostatic behavior, wound closure, histological evaluation, immunohistochemical analysis, and serum cytokine measurements—were newly generated in the present study. For these experiments, the hydrogels were freshly prepared using the same predefined composition and standardized preparation procedure. Reproducibility of hydrogel preparation was maintained through fixed CMC, HA, and RGD concentrations, defined EDC/NHS-assisted gelation conditions, standardized mixing and processing parameters, and consistent post-gelation washing and sterilization. Formation of a stable, non-flowing hydrogel was used as the operational criterion for successful gelation before biological testing.

Thus, the information summarized in this section establishes the identity and structural continuity of the material with the previously characterized hydrogel system, while the subsequent sections report newly generated functional and in vivo biological data.

### 2.2. Antibacterial Activity of CMC-Based Hydrogels

The antibacterial activity of different CMC-based hydrogels was evaluated against *E. coli* and *S. aureus* using the agar diffusion method. The primary purpose of this experiment was to determine whether each hydrogel formulation exhibited detectable antibacterial activity under the assay conditions, rather than to quantify bactericidal potency or establish an absolute ranking of antibacterial efficacy.

**Figure 2:**
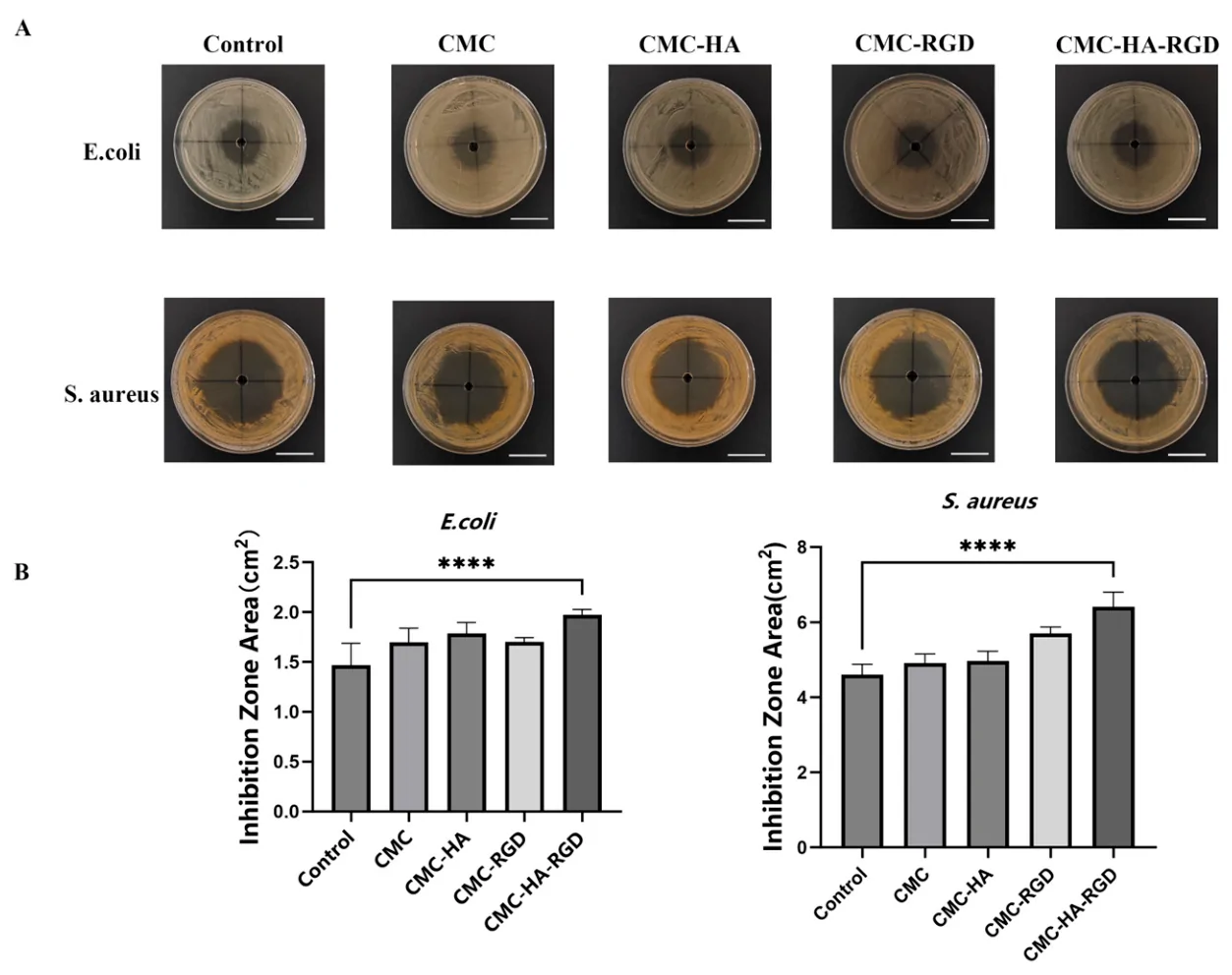
Antibacterial activity of CMC-based hydrogels. (**A**) Representative images of inhibition zones formed by different CMC-based hydrogels against *E. coli* and *S. aureus*. (**B**) Quantitative analysis of inhibition zone areas. The left panel represents the inhibition zone areas against *E. coli,* and the right panel represents those against *S. aureus*. The positive control group was treated with penicillin/streptomycin (PNS) without hydrogel application. The CMC group received CMC hydrogel treatment; the CMC-HA group received CMC hydrogel containing 0.9% HA; the CMC-RGD group received CMC hydrogel containing 0.02 mg/mL RGD peptides; and the CMC-HA-RGD group received CMC hydrogel containing both 0.9% HA and 0.02 mg/mL RGD peptides. Scale bar = 1 cm. Data are presented as mean ± standard deviation (SD). **** *p* < 0.0001.

As shown in Figure 2A, all CMC-based hydrogel formulations produced visible inhibition zones against both tested bacterial species, indicating that each formulation exhibited detectable in vitro antibacterial activity under the agar-diffusion conditions used in this study. The CMC-HA-RGD formulation produced relatively larger inhibition zones than the other hydrogel groups.

Quantitative measurement of the inhibition-zone areas further documented these observations (Figure 2B). Against *E. coli*, the CMC-HA-RGD hydrogel produced an inhibition-zone area of approximately 2.0 cm^2^, representing the largest value among the tested formulations. Similarly, against *S. aureus*, the CMC-HA-RGD formulation produced the largest inhibition-zone area, reaching approximately 6.0 cm^2^. All four hydrogel formulations showed larger inhibition zones against *S. aureus* than against *E. coli*, indicating a species-dependent difference in the observed agar-diffusion response.

Because inhibition-zone formation in hydrogel systems can be influenced not only by antibacterial effects but also by the release and diffusion of soluble or releasable components through the agar matrix, differences in inhibition-zone area were interpreted as differences in agar-diffusion response rather than as direct quantitative differences in intrinsic bactericidal activity. Thus, the principal finding of this assay is that all four CMC-based hydrogel formulations exhibited detectable in vitro antibacterial activity against both tested bacterial species. The larger inhibition zones observed for CMC-HA-RGD indicate a greater agar-diffusion response under the present experimental conditions, but are not interpreted as evidence of superior intrinsic bactericidal potency.

Overall, these findings confirm that CMC, CMC-HA, CMC-RGD, and CMC-HA-RGD all exhibited antibacterial activity under agar-diffusion conditions, supporting the antibacterial potential of the CMC-based hydrogel system for wound-dressing applications.

### 2.3. In Vivo Hemostatic Performance of CMC-Based Hydrogels

**Figure 3:**
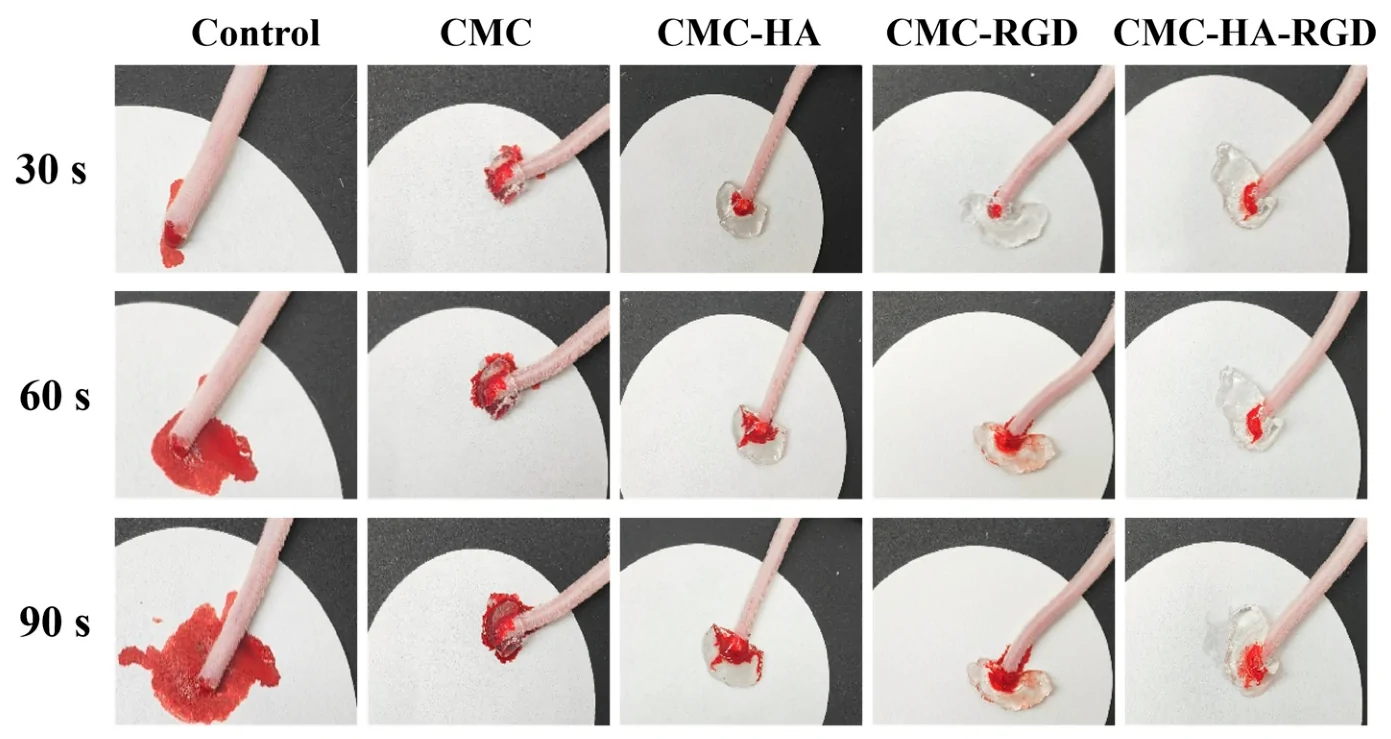
In vivo hemostatic performance of CMC-based hydrogel in a mouse tail transection model. Representative images showing the hemostatic process of different CMC-based hydrogels at 30, 60, and 90 s after tail transection. The control group received tail transection without hydrogel treatment. The CMC group received CMC hydrogel treatment; the CMC-HA group received CMC hydrogel containing 0.9% HA; the CMC-RGD group received CMC hydrogel containing 0.02 mg/mL RGD peptides; and the CMC-HA-RGD group received CMC hydrogel containing both 0.9% HA and 0.02 mg/mL RGD peptides.

The in vivo hemostatic behavior of the different CMC-based hydrogel formulations was evaluated using a mouse tail-transection model. As shown in Figure 3, the control group without hydrogel treatment exhibited persistent visible bleeding throughout the observation period. In contrast, all hydrogel-treated groups showed a progressive reduction in visible bleeding over time.

Compared with the CMC group, the functionalized hydrogel groups generally showed less visible bleeding during the observation period. At 30 s, limited visible bleeding was observed in the CMC-RGD group, while at 60 and 90 s, both the CMC-RGD and CMC-HA-RGD hydrogels showed only limited visible bleeding. The CMC-HA group also showed a reduction in visible bleeding compared with the CMC group.

These observations indicate favorable hemostatic behavior of the HA- and/or RGD-containing CMC hydrogel formulations. In particular, the RGD-containing formulations showed a pronounced reduction in visible bleeding during the observation period. The combined incorporation of HA and RGD in the CMC-HA-RGD formulation was also associated with reduced visible bleeding. Because the hemostatic assessment was based on visual observations at predefined time points (30, 60, and 90 s), these findings are interpreted qualitatively. Overall, the tail-transection results support the favorable in vivo hemostatic behavior of the functionalized CMC-based hydrogel formulations.

### 2.4. CMC-HA-RGD Hydrogel Accelerated Wound Closure In Vivo

**Figure 4:**
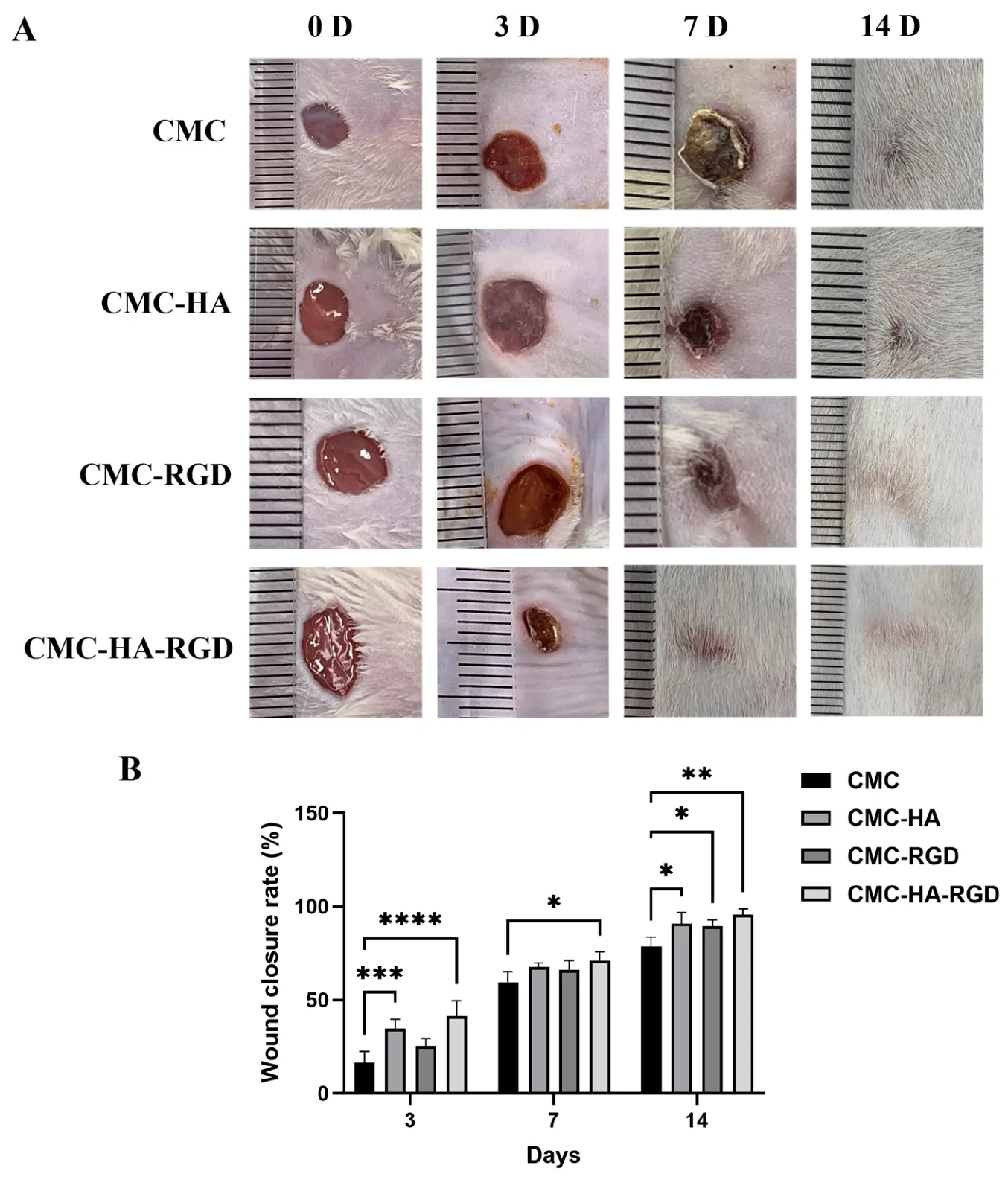
Effect of CMC-based hydrogels on wound healing in a mouse full-thickness skin defect model. (**A**) Representative images of wounds from different treatment groups at days 0, 3, 7, and 14 after treatment. The ruler shown beside each wound was used as the spatial reference; the smallest ruler graduation represents 1 mm. Wound images were individually calibrated using this 1 mm reference before quantitative area measurement. (**B**) Quantitative analysis of wound area percentage during the healing process. The CMC group received CMC hydrogel treatment; the CMC-HA group received CMC hydrogel containing 0.9% HA; the CMC-RGD group received CMC hydrogel containing 0.02 mg/mL RGD peptides; and the CMC-HA-RGD group received CMC hydrogel containing both 0.9% HA and 0.02 mg/mL RGD peptides. Data are presented as mean ± SD (*n* = 5 independent biological replicates per treatment group per time point). Statistical analysis was performed using two-way ANOVA with hydrogel treatment and time as factors, followed by Dunnett’s multiple-comparisons test for comparisons of the CMC-HA, CMC-RGD, and CMC-HA-RGD hydrogels with the CMC reference group within each time point. * *p* < 0.05, ** *p* < 0.01, *** *p* < 0.001, **** *p* < 0.0001.

The wound healing performance of different CMC-based hydrogels was evaluated using a full-thickness skin defect model in mice. As shown in Figure 4A, wound size gradually decreased in all groups during the 14-day observation period, while differences in wound contraction were observed among the hydrogel formulations. Compared with the CMC hydrogel group, both CMC-HA and CMC-RGD treatments resulted in higher wound closure rates, suggesting that the incorporation of HA or RGD improved the wound-repair performance of the CMC matrix. The CMC-HA-RGD group exhibited the smallest residual wound area throughout the healing process, with more complete wound closure observed at day 14.

Quantitative analysis showed that the wound closure rates of the CMC, CMC-HA, CMC-RGD, and CMC-HA-RGD hydrogels were approximately 16.47%, 33.26%, 25.14%, and 41.39% on day 3, respectively. These values increased to approximately 58.18%, 67.39%, 66.31%, and 71.18% on day 7, and further reached 79.85%, 90.68%, 87.43%, and 95.56% on day 14, respectively. The CMC-HA-RGD group showed the highest wound closure rate among the tested formulations at all examined time points, reaching approximately 95.56% closure by day 14.

Following the combined incorporation of HA and RGD, the CMC-HA-RGD group exhibited a higher wound closure rate, indicating that differences in hydrogel composition were associated with improved wound-repair performance. Overall, among the tested formulations, the CMC-HA-RGD hydrogel achieved the highest wound closure rate (95.56%) and the smallest residual wound area at day 14, indicating favorable wound-repair performance in this mouse full-thickness skin wound model.

### 2.5. Histological Evaluation

**Figure 5:**
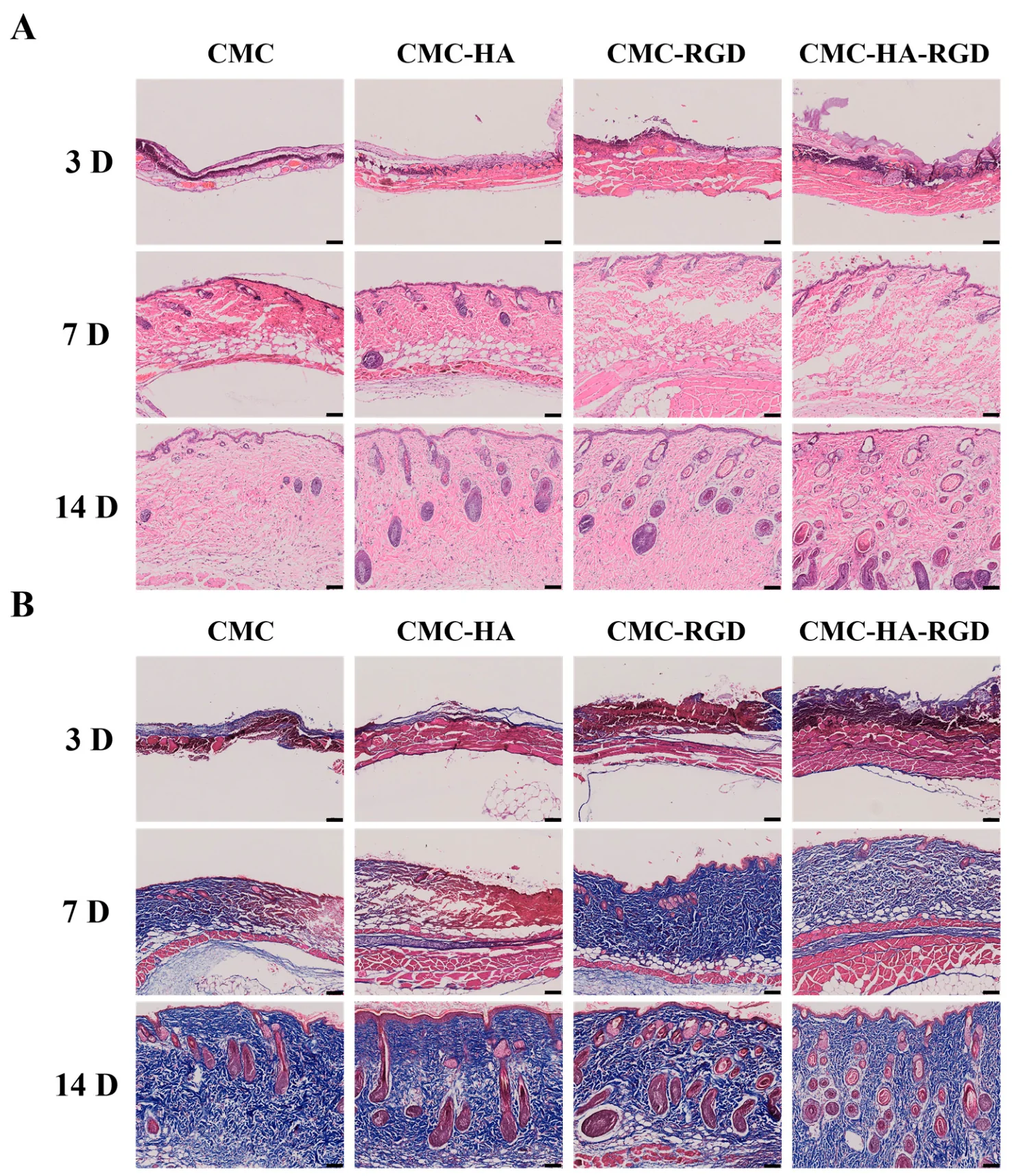
Histological evaluation of wound tissue regeneration after treatment with CMC-based hydrogels. (**A**) Hematoxylin and eosin (H&E) staining of wound tissues collected at days 3, 7, and 14 after treatment, showing the morphological changes, inflammatory response, and epidermal regeneration in different groups. (**B**) Masson’s trichrome staining of wound tissues at different healing stages, showing collagen deposition and extracellular matrix remodeling. The CMC group received CMC hydrogel treatment; the CMC-HA group received CMC hydrogel containing 0.9% HA; the CMC-RGD group received CMC hydrogel containing 0.02 mg/mL RGD peptides; and the CMC-HA-RGD group received CMC hydrogel containing both 0.9% HA and 0.02 mg/mL RGD peptides. Scale bar = 100 μm.

To further assess the quality of wound repair, H&E and Masson’s trichrome staining were performed on wound tissues collected on days 3, 7, and 14 after treatment. As shown in Figure 5, the histological appearance of all groups changed markedly with the progression of healing, with gradual restoration of the epidermis, formation of dermal tissue, and accumulation of collagen fibers. However, the extent of tissue regeneration differed among the hydrogel formulations. Compared with the CMC group, the CMC-HA, CMC-RGD, and CMC-HA-RGD hydrogels showed earlier improvement in tissue organization, and the differences became more evident at the later stage of healing.

H&E staining showed that the wound tissues remained incompletely regenerated on day 3, with discontinuous epidermal structures and immature tissue organization in all groups (Figure 5A). At this stage, the CMC group showed a relatively thin and discontinuous newly formed epidermal layer, whereas the CMC-HA and CMC-RGD groups exhibited more continuous tissue coverage. The CMC-HA-RGD group showed a relatively more compact tissue structure at the wound site, indicating an earlier progression of tissue repair. By day 7, epidermal regeneration and dermal tissue formation were more apparent in all groups. Compared with the CMC group, the CMC-HA and CMC-RGD groups showed more organized dermal structures, while the CMC-HA-RGD group exhibited a more continuous epidermal layer and a denser regenerated tissue structure. By day 14, the differences among groups were more pronounced. The CMC group showed relatively incomplete dermal organization and fewer recognizable skin appendage structures, whereas the CMC-HA and CMC-RGD groups exhibited more complete skin architecture with increased numbers of follicle-like structures. Notably, the CMC-HA-RGD group showed a well-developed epidermal–dermal structure accompanied by abundant skin appendage-like structures, suggesting a more advanced level of tissue regeneration.

Masson’s trichrome staining further demonstrated a time-dependent increase in collagen deposition in the regenerated tissue (Figure 5B). On day 3, collagen fibers were relatively sparse and mainly distributed in a discontinuous manner in all groups. By day 7, blue-stained collagen areas became more prominent, particularly in the functionalized hydrogel groups, indicating enhanced extracellular matrix formation. At day 14, the CMC group still showed relatively loose collagen distribution, whereas the CMC-HA and CMC-RGD groups exhibited denser collagen deposition and a more organized fibrous structure. The CMC-HA-RGD group displayed the most pronounced collagen accumulation, with dense and relatively well-organized blue-stained collagen fibers extending throughout the regenerated dermal tissue. The presence of more mature collagen-rich tissue together with the development of skin appendage-like structures further indicated improved dermal reconstruction in this group [47,48].

Taken together, the H&E and Masson’s trichrome results showed that incorporation of HA or RGD into the CMC hydrogel promoted epidermal regeneration, dermal reconstruction, and collagen deposition compared with the CMC hydrogel alone. Among the tested formulations, the CMC-HA-RGD hydrogel exhibited more favorable histological features at the later stage of healing, characterized by a more complete epidermal–dermal structure, more abundant skin appendage-like structures, and denser collagen deposition. These findings indicate that the CMC-HA-RGD formulation was associated with enhanced tissue regeneration and collagen deposition in this mouse full-thickness skin wound model, supporting its potential as a multifunctional material for skin wound repair.

### 2.6. CMC-HA-RGD Hydrogel Regulated Inflammatory Responses and Collagen Remodeling During Wound Healing

**Figure 6:**
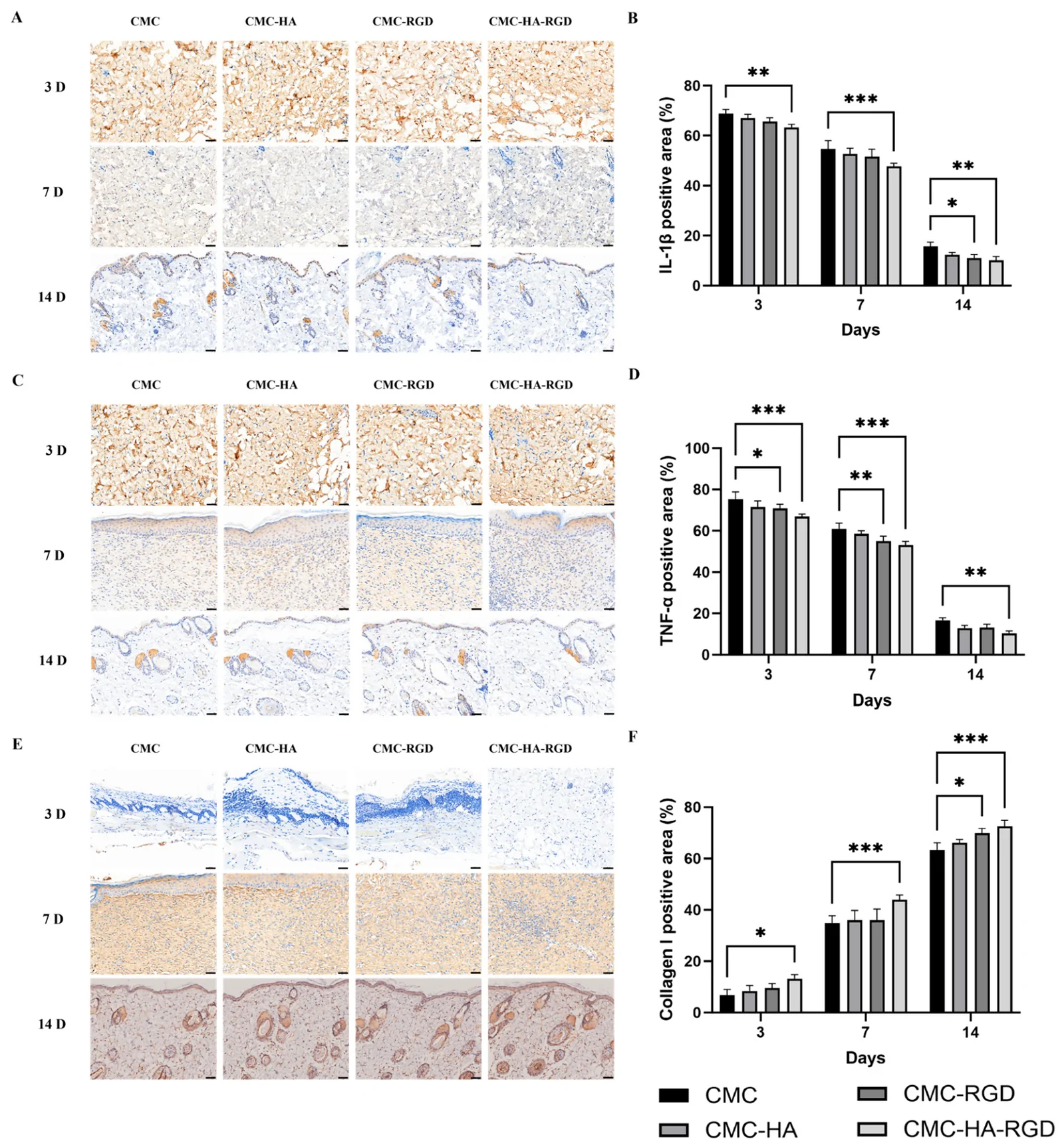
Immunohistochemical analysis of inflammatory cytokine expression and collagen remodeling in wound tissues after treatment with CMC-based hydrogels. Representative immunohistochemical staining and quantitative analysis of IL-1β, TNF-α, and collagen I expression in wound tissues collected at days 3, 7, and 14 after treatment. (**A**,**B**) Representative images and quantitative analysis of IL-1β expression. (**C**,**D**) Representative images and quantitative analysis of TNF-α expression. (**E**,**F**) Representative images and quantitative analysis of collagen I expression. The CMC group received CMC hydrogel treatment; the CMC-HA group received CMC hydrogel containing 0.9% HA; the CMC-RGD group received CMC hydrogel containing 0.02 mg/mL RGD peptides; and the CMC-HA-RGD group received CMC hydrogel containing both 0.9% HA and 0.02 mg/mL RGD peptides. Scale bar = 50 μm. Data are presented as mean ± SD (*n* = 5 independent biological replicates per treatment group per time point). Statistical analysis was performed using two-way ANOVA with hydrogel treatment and time as factors, followed by Dunnett’s multiple-comparisons test for comparisons of the CMC-HA, CMC-RGD, and CMC-HA-RGD hydrogels with the CMC reference group within each time point. * *p* < 0.05, ** *p* < 0.01, *** *p* < 0.001.

Immunohistochemical staining was performed at days 3, 7, and 14 to evaluate the positive staining areas of the inflammatory markers interleukin-1β (IL-1β) and tumor necrosis factor-α (TNF-α) and the extracellular matrix-associated protein collagen I during wound healing. As shown in Figure 6, the positive staining areas of IL-1β and TNF-α progressively decreased from day 3 to day 14, whereas collagen I showed an overall increasing trend over the same period. The magnitude of these changes differed among the hydrogel formulations.

At day 3, the IL-1β-positive areas were 68.88%, 66.97%, 65.64%, and 63.32% in the CMC, CMC-HA, CMC-RGD, and CMC-HA-RGD hydrogels, respectively. TNF-α showed a similar pattern, with positive areas of 75.21%, 71.61%, 70.84%, and 66.86%, respectively. Thus, incorporation of HA or RGD partially reduced the early inflammatory response, while the CMC-HA-RGD hydrogel showed the lowest positive staining areas for both inflammatory markers. As healing progressed, the differences became more apparent. By day 14, the IL-1β-positive areas decreased to 15.72% in the CMC group, compared with 12.36%, 11.06%, and 10.16% in the CMC-HA, CMC-RGD, and CMC-HA-RGD hydrogels, respectively. Similarly, TNF-α-positive areas decreased to 16.70%, 12.91%, 13.14%, and 10.40%, respectively. Compared with the CMC hydrogel, the CMC-HA-RGD hydrogel reduced the IL-1β- and TNF-α-positive areas by approximately 35.37% and 37.74%, respectively, at day 14, indicating more effective attenuation of the local inflammatory response during the later stage of wound healing.

In contrast, collagen I-positive areas increased markedly during wound repair. At day 3, the collagen I-positive areas were 6.77%, 8.43%, 9.54%, and 13.21% in the CMC, CMC-HA, CMC-RGD, and CMC-HA-RGD hydrogels, respectively. By day 7, these values increased to 41.62%, 35.99%, 36.04%, and 43.95%, respectively. At day 14, the values further increased to 63.36%, 66.15%, 69.83%, and 72.58%, respectively. Notably, the CMC-HA-RGD group showed the highest collagen I-positive area at all three time points, with a particularly clear advantage at days 7 and 14. At day 14, the collagen I-positive area in the CMC-HA-RGD group reached 72.58%, compared with 63.36% in the CMC hydrogel, indicating enhanced collagen deposition during the later stage of tissue repair.

Taken together, the immunohistochemical results showed that incorporation of HA or RGD into the CMC hydrogel was associated with reduced IL-1β and TNF-α expression and increased collagen I deposition compared with the CMC hydrogel alone. At day 14, the CMC-HA-RGD group exhibited an IL-1β-positive area of 10.16% (*p* = 0.0042), a TNF-α-positive area of 10.40% (*p* = 0.0034), and a collagen I-positive area of 72.58% (*p* = 0.0005). These findings indicate that the CMC-HA-RGD formulation was associated with lower levels of the examined inflammatory markers and enhanced collagen deposition during wound repair. Combined with the histological observations, these findings provide further evidence of tissue repair in the CMC-HA-RGD hydrogel during the later stage of wound healing.

### 2.7. CMC-HA-RGD Hydrogel Attenuated Inflammatory Responses During Wound Healing

**Figure 7:**
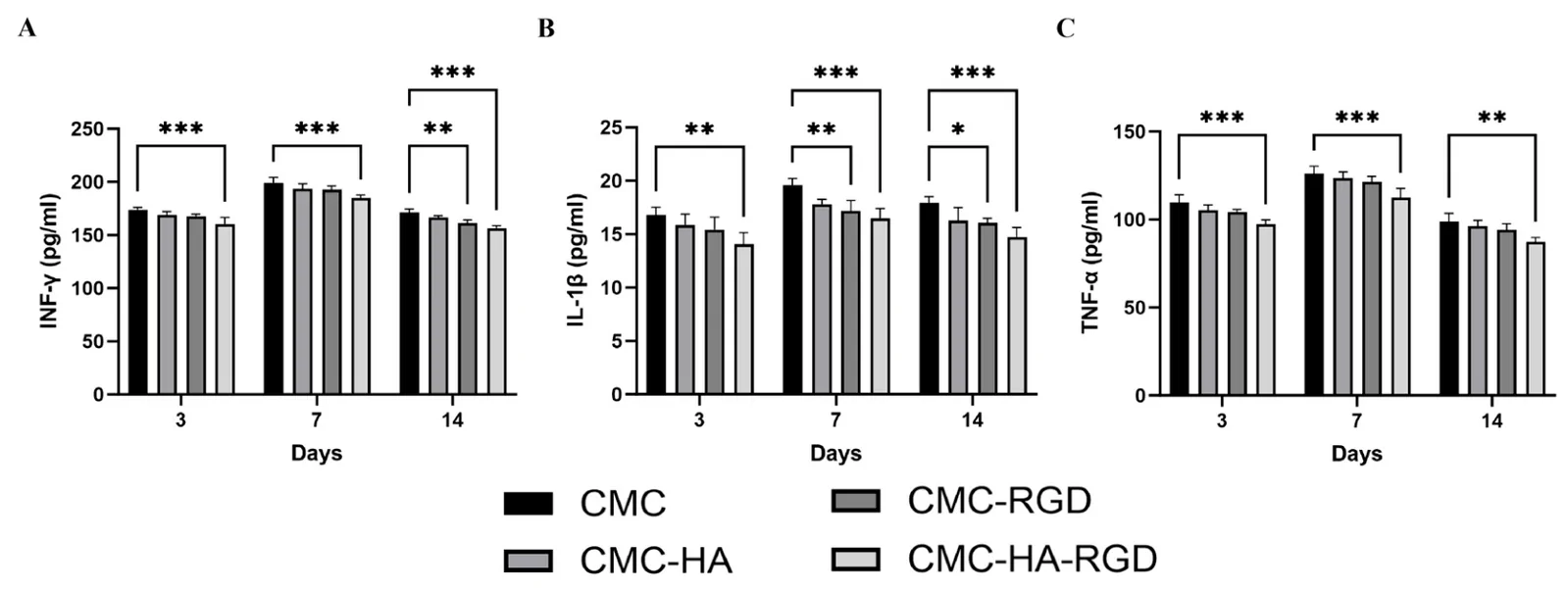
Effects of CMC-based hydrogels on serum inflammatory cytokine levels during wound healing. Serum levels of inflammatory cytokines were measured by ELISA at days 3, 7, and 14 after treatment. (**A**) IFN-γ levels. (**B**) IL-1β levels. (**C**) TNF-α levels. The CMC group received CMC hydrogel treatment; the CMC-HA group received CMC hydrogel containing 0.9% HA; the CMC-RGD group received CMC hydrogel containing 0.02 mg/mL RGD peptides; and the CMC-HA-RGD group received CMC hydrogel containing both 0.9% HA and 0.02 mg/mL RGD peptides. Data are presented as mean ± standard deviation (SD) (*n* = 5 independent biological replicates per treatment group per time point). Statistical analysis was performed using two-way ANOVA with hydrogel treatment and time as factors, followed by Dunnett’s multiple-comparisons test for comparisons of the CMC-HA, CMC-RGD, and CMC-HA-RGD hydrogels with the CMC reference group within each time point. * *p* < 0.05, ** *p* < 0.01, *** *p* < 0.001.

To evaluate the anti-inflammatory effects of different CMC-based hydrogels during wound healing, serum levels of interferon-γ (IFN-γ), IL-1β, and TNF-α were measured by ELISA on days 3, 7, and 14 after treatment. As shown in Figure 7, all three inflammatory cytokines exhibited a similar temporal pattern in the four hydrogel-treated groups, with serum levels increasing from day 3 to day 7 and subsequently decreasing by day 14. The increase was more pronounced on day 7, whereas all three cytokines showed a clear decline by day 14. For example, serum IFN-γ levels in the CMC, CMC-HA, CMC-RGD, and CMC-HA-RGD hydrogels increased from 173.82, 168.47, 166.35, and 159.74 pg/mL on day 3 to 198.63, 193.16, 191.27, and 184.52 pg/mL, respectively, on day 7, followed by a decrease on day 14. IL-1β and TNF-α showed similar temporal changes, with higher serum levels during the middle stage of wound healing and lower levels at the later stage.

Comparison among the four hydrogel groups showed that the CMC-HA-RGD group consistently exhibited the lowest serum levels of IFN-γ, IL-1β, and TNF-α at all examined time points. Both CMC-HA and CMC-RGD treatments resulted in lower cytokine levels compared with the CMC hydrogels. This difference was particularly evident on day 14, when the serum levels of IFN-γ, IL-1β, and TNF-α in the CMC-HA-RGD hydrogel decreased to 155.83, 14.61, and 86.54 pg/mL, respectively, compared with 171.38, 17.82, and 98.17 pg/mL in the CMC hydrogel, corresponding to reductions of 9.08%, 18.01%, and 11.85%, respectively.

These results indicate that HA- or RGD-containing formulations were associated with reduced serum inflammatory cytokine levels during wound healing. The lower serum levels of IL-1β and TNF-α in the CMC-HA-RGD hydrogel were consistent with the reduced positive staining areas observed by immunohistochemistry, suggesting similar changes in local and systemic inflammatory markers. Overall, among the tested formulations, the CMC-HA-RGD hydrogel showed lower levels of the examined inflammatory cytokines, which were accompanied by improved wound closure and tissue repair outcomes observed in this study.

## 3. Discussion

The present study extends our previous characterization of the CMC-HA-RGD hydrogel by evaluating its antibacterial activity, hemostatic performance, and therapeutic effects in a full-thickness wound model. Among the four CMC-based formulations, the simultaneous incorporation of HA and RGD produced the most favorable overall performance. The CMC-HA-RGD hydrogel achieved wound closure rates of 41.39%, 71.18%, and 95.56% on days 3, 7, and 14, respectively. At day 14, the closure rate was 15.71, 4.88, and 8.13 percentage points higher than those of the CMC, CMC-HA, and CMC-RGD hydrogels, respectively. This result is consistent with our previous in vitro study, in which the same formulation promoted fibroblast viability, proliferation, and migration [46]. Moreover, the final wound closure rate was comparable with that reported for other functional CMC-based hydrogels; for example, a carboxymethyl chitosan/oxidized sodium alginate hydrogel containing bioactive peptides achieved approximately 97.95% wound healing at day 14 [49]. Although differences in animal models and formulations preclude direct equivalence, these results place the wound-healing performance of CMC-HA-RGD within the range reported for advanced CMC-based wound dressings.

The antibacterial results further demonstrated functional differences among the hydrogel formulations. The CMC-HA-RGD hydrogel produced inhibition-zone areas of approximately 2.0 cm^2^ against *E. coli* and 6.0 cm^2^ against *S. aureus*, representing the largest inhibition-zone areas among the tested formulations. The larger inhibition zone observed against *S. aureus* than against *E. coli* indicates a species-dependent difference in antibacterial response, and similar differences have been reported for chitosan and its derivatives [50]. One proposed antibacterial mechanism of chitosan-based materials involves electrostatic interactions between their amino groups and negatively charged bacterial cell surfaces, which may affect bacterial membrane integrity and permeability [51,52]. The larger inhibition zones observed for the CMC-HA-RGD formulation may also be associated with changes in hydrogel network properties, component diffusion behavior, and material–bacteria interactions following the incorporation of HA and RGD. Importantly, the agar-diffusion assay used in the present study was designed to provide a quantitative comparison of growth-inhibition responses among the four CMC-based hydrogel formulations under identical experimental conditions. The CMC-only hydrogel served as the base-matrix reference, enabling formulation-dependent differences associated with HA and/or RGD incorporation to be evaluated within the same CMC hydrogel system. Accordingly, the present findings are interpreted specifically as evidence of in vitro antibacterial activity under agar-diffusion conditions rather than as evidence of a defined direct contact-killing mechanism. Overall, these findings support the antibacterial potential of the CMC-HA-RGD hydrogel for wound-dressing applications. Complementary viable-count or direct contact-based assays may further characterize its antibacterial mode of action in future studies.

The improvement in wound closure was accompanied by changes in histological features and inflammatory markers. On day 14, histological observations showed a relatively more complete epidermal–dermal architecture, more abundant skin appendage-like structures, and denser collagen deposition in the CMC-HA-RGD group than in the other tested CMC-based hydrogels. At the same time point, the IL-1β- and TNF-α-positive areas in the CMC-HA-RGD group were 10.16% and 10.40%, respectively, whereas the collagen I-positive area reached 72.58%. Compared with the CMC group, the IL-1β- and TNF-α-positive areas were approximately 35.37% and 37.74% lower, respectively. Serum IFN-γ, IL-1β, and TNF-α levels showed changes in the same direction as the local immunohistochemical findings, further indicating lower levels of the examined inflammatory markers in the CMC-HA-RGD group during the later stage of wound repair. Cutaneous wound healing involves interconnected inflammatory, proliferative, and remodeling processes, in which dynamic changes in inflammatory mediators, collagen deposition, and extracellular matrix remodeling are important features of tissue repair [3,53]. In the present study, the concurrent presence of lower levels of the examined inflammatory markers, a higher collagen I-positive area, and more favorable histological features was consistent with tissue reconstruction during the later stage of wound repair.

The mouse tail-transection experiment showed favorable hemostatic behavior of the RGD-containing hydrogel formulations. At 30 s, relatively limited visible bleeding was observed in the CMC-RGD group, while at 60 and 90 s, only limited visible bleeding was observed in both the CMC-RGD and CMC-HA-RGD hydrogels, indicating favorable hemostatic performance of both RGD-containing formulations during the observation period. Similar hemostatic performance has also been reported in other related hydrogel studies. For example, Zhai et al. developed a supramolecular hydrogel by co-assembling an RGD-containing cell-adhesive peptide conjugate (Pept-1) with alginate, which exhibited an in vitro clotting time of approximately 41 s and reduced blood loss to approximately 18% of that in the untreated control in a mouse liver-puncture model [54]. Although the material composition and hemostatic model differed from those used in the present study, this report provides relevant literature context for the hemostatic behavior observed in our hydrogel formulations.

Despite the favorable overall performance of CMC-HA-RGD across multiple evaluated endpoints, several limitations should be acknowledged. First, because no factorial interaction analysis was performed, the present findings do not establish a synergistic interaction between HA and RGD. Second, antibacterial activity was evaluated only in vitro, whereas hemostatic performance was primarily assessed by visual observations at predefined time points; therefore, in vivo infection control and quantitative hemostatic performance require further validation. In addition, the observed changes in inflammatory markers and collagen I reflect biological changes associated with wound repair but are insufficient to establish specific molecular mechanisms. Future studies incorporating more clinically relevant wound models, quantitative hemostatic assessments, and targeted mechanistic investigations of relevant signaling pathways, such as integrin-related signaling, may help further clarify the biological basis of CMC-HA-RGD activity and provide a more comprehensive foundation for its future translational evaluation.

Overall, among the tested formulations, CMC-HA-RGD exhibited a broad functional profile characterized by in vitro antibacterial activity, favorable hemostatic behavior, higher wound closure, lower levels of the examined inflammatory markers, and greater collagen deposition together with improved histological repair features. These findings suggest that the wound-repair performance of CMC-HA-RGD may involve multiple material-related and biological effects, although the underlying mechanisms and the individual and combined contributions of HA and RGD remain to be further elucidated. Collectively, the present findings support the potential of CMC-HA-RGD as a multifunctional material for skin wound repair and provide an experimental basis for further mechanistic investigation and translational evaluation.

## 4. Materials and Methods

### 4.1. Chemicals and Reagents

Carboxymethyl chitosan (CMC, CAS No. 83512-85-0, degree of carboxymethylation ≥ 80%, viscosity 200–300 cps; average polymer molecular weight not specified by the supplier), RGD peptide (RGD, CAS No. 99896-85-2, purity 99.60%, molecular weight 346.36 g/mol), 1-ethyl-3-(3-dimethylaminopropyl) carbodiimide (EDC, CAS No. 25952-53-8), and N-hydroxysuccinimide (NHS, CAS No. 6066-82-6) were purchased from Sigma-Aldrich (St. Louis, MO, USA). Hyaluronic acid (HA, CAS No. 9004-61-9, purity ≥ 97%, medium-high-molecular-weight grade, average molecular weight 1.0–1.3 MDa) was obtained from Macklin (Shanghai, China). *Escherichia coli* (*E. coli*) and *Staphylococcus aureus* (*S. aureus*) strains were provided by the laboratory of Shanghai Ocean University. Agar and Luria–Bertani (LB) broth were purchased from Qingdao Haibo Biotechnology Co., Ltd. (Qingdao, China). Penicillin/streptomycin antibiotic solution was obtained from Beijing Lanjieke Technology Co., Ltd. (Beijing, China). Xylene and glacial acetic acid were purchased from Sinopharm Chemical Reagent Co., Ltd. (Shanghai, China). Hematoxylin solution, eosin solution, saturated lithium carbonate solution, Masson’s trichrome staining kit, Bouin’s fixative, and neutral balsam were obtained from Zhuhai Beso Biotechnology Co., Ltd. (Zhuhai, China). Environmentally friendly dewaxing solution, citrate antigen retrieval solution, and phosphate-buffered saline (PBS) were purchased from Wuhan Servicebio Technology Co., Ltd. (Wuhan, China). Antibodies against IL-1β, TNF-α, and collagen I were purchased from Wuhan Servicebio Technology Co., Ltd. ELISA kits for IFN-γ, IL-1β, and TNF-α were obtained from Shanghai Jianglai Biotechnology Co., Ltd. (Shanghai, China). The relevant physicochemical properties and specifications of CMC, HA, and RGD are summarized in Table 1.

### 4.2. Preparation of CMC-Based Hydrogels

The hydrogel formulations used in the present study were prepared according to our previously reported method [46]. Briefly, 0.1 g of CMC was dissolved in 10 mL of distilled water under continuous magnetic stirring at 600 rpm and 37 °C to obtain a 1 wt% CMC precursor solution. The EDC/NHS crosslinking system, consisting of 10 μL EDC and 0.05 g NHS, was pre-mixed at 25 °C for 30 min before use. For the CMC formulation, the CMC precursor solution was directly combined with the pre-activated EDC/NHS system. For the CMC-HA formulation, aqueous HA was first mixed with the CMC precursor solution to a final concentration of 0.9 wt%, followed by addition of the EDC/NHS crosslinking system. For the CMC-RGD formulation, aqueous RGD peptide was introduced into the CMC precursor solution to a final concentration of 0.02 mg/mL before addition of EDC/NHS. For the CMC-HA-RGD formulation, both HA and RGD were incorporated into the CMC precursor solution to final concentrations of 0.9 wt% and 0.02 mg/mL, respectively, prior to addition of the crosslinking system.

Following addition of EDC/NHS, the precursor mixtures were allowed to react and gel under the established conditions until stable, non-flowing hydrogels were formed. Initial gelation occurred within approximately 20–30 min. The formed hydrogels were then maintained under the same conditions to allow complete gel formation and stabilization of the three-dimensional network.

After gelation, the hydrogels were thoroughly washed with 100 mM PBS (pH 7.4), followed by distilled water, to remove unreacted EDC/NHS, water-soluble EDC-derived urea products, hydrolyzed NHS species, and other unreacted soluble components. For subsequent biological experiments, the hydrogels were sterilized by UV-C irradiation at 254 nm for 1 h and subsequently rinsed three times with sterile PBS before use. As residual EDC/NHS concentrations were not quantitatively determined, repeated washing was employed to minimize residual crosslinking reagents prior to biological evaluation. The resulting hydrogels were collected and used for subsequent antibacterial, hemostatic, and in vivo wound-healing experiments.

The final formulations were prepared as follows: CMC hydrogel (1 wt% CMC), CMC-HA hydrogel (1 wt% CMC + 0.9 wt% HA), CMC-RGD hydrogel (1 wt% CMC + 0.02 mg/mL RGD), and CMC-HA-RGD hydrogel (1 wt% CMC + 0.9 wt% HA + 0.02 mg/mL RGD).

### 4.3. Antibacterial Activity Assessment

The antibacterial activity of the CMC-based hydrogel formulations was evaluated against *E. coli* and *S. aureus* using the agar diffusion method [55]. *E. coli* and *S. aureus* were laboratory-maintained isolates obtained from a collaborating laboratory at Shanghai Ocean University and subsequently cultured in our laboratory. Specific strain accession numbers were not available for these laboratory-provided isolates. Briefly, bacterial strains were cultured in LB broth at 37 °C overnight and diluted to obtain bacterial suspensions with a concentration of approximately 1.0 × 10^6^ CFU/mL. The bacterial suspensions were uniformly spread onto LB agar plates to form a bacterial lawn. Wells with identical diameters were prepared in the agar plates, and 100 μL of different hydrogel formulations, including CMC, CMC-HA, CMC-RGD, and CMC-HA-RGD hydrogels, were added into the wells. The CMC hydrogel was used as the base-matrix reference for comparison with the HA- and/or RGD-containing formulations, whereas penicillin/streptomycin antibiotic solution (PNS) was used as the positive antibacterial control. The plates were incubated at 37 °C for 24 h. After incubation, the inhibition zones formed around each sample were photographed. The image scale was calibrated using the known scale reference (1 cm), and the inhibition-zone areas were subsequently quantified using ImageJ 1.51n software. Antibacterial performance was evaluated quantitatively based on the inhibition-zone area of each treatment group under identical experimental conditions. All experiments were independently performed in triplicate.

### 4.4. Tail Amputation Hemostasis Model

Six-week-old male SPF-grade Kunming mice were obtained from Spevo (Suzhou) Biotechnology Co., Ltd., Suzhou, China, and housed under standard laboratory conditions with a 12 h light/dark cycle, controlled temperature and humidity, and free access to food and water. All mice were acclimatized for one week before the experiment.

Before the procedure, mice were anesthetized, and the distal portion of the tail was cleaned and disinfected under sterile conditions. A standardized tail transection was performed at a predetermined position [56]. After tail amputation, mice were randomly divided into five groups (*n* = 3 per group): control group, CMC hydrogel group, CMC-HA hydrogel group, CMC-RGD hydrogel group, and CMC-HA-RGD hydrogel group. Immediately after transection, the bleeding wounds were treated with the corresponding hydrogel formulations, while mice in the control group received no hydrogel treatment. The bleeding process was continuously monitored, and the hemostatic status was recorded at predetermined time points (0, 30, 60, and 90 s). Standardized photographs were captured to document the bleeding conditions. Hemostasis was considered achieved when visible bleeding completely ceased without further blood leakage. The hemostatic performance of each treatment group was evaluated by comparing the bleeding cessation time and hemostatic status among different groups. All experiments were conducted under identical experimental conditions.

All animal procedures were conducted in accordance with the guidelines and regulations outlined in the Scientific Ethics in the Care and Use of Laboratory Animals of Shanghai Ocean University. The experimental protocol was approved by the Institutional Animal Care and Use Committee (License No.: SHOU-DW-2025-230).

### 4.5. Establishment of Full-Thickness Skin Wound Model

Before surgical procedures, mice were anesthetized. The dorsal hair was carefully removed using an electric shaver, and the surgical area was disinfected. A circular full-thickness excisional wound with a diameter of 8 mm was created on the dorsal skin of each mouse under aseptic conditions [57,58]. After wound establishment, mice were randomly divided into four groups (*n* = 15 per group): CMC hydrogel group; CMC-HA hydrogel; CMC-RGD hydrogel; and CMC-HA-RGD hydrogel. The corresponding hydrogel formulation was directly applied to the wound site of each mouse. The wounds were photographed at predetermined time points (days 0, 3, 7, and 14), using the same millimeter-graduated ruler as an internal spatial reference in each photograph. The smallest ruler graduation corresponded to 1 mm. For quantitative analysis, each image was individually calibrated in ImageJ using the 1 mm ruler graduation as the known distance, after which the wound boundary was delineated and the wound area was quantified. This image-specific calibration accounted for minor differences in image magnification resulting from variations in the camera-to-wound distance during photography.

For terminal sampling, five mice from each treatment group were assigned to each of the three predefined endpoints (days 3, 7, and 14). One mouse was considered one independent biological replicate; therefore, each treatment group comprised *n* = 5 biological replicates at each terminal time point. At the designated endpoint, the corresponding animals were euthanized, and wound tissues and blood samples were collected for subsequent histological, immunohistochemical, and serum cytokine analyses.

The wound-healing dataset was evaluated according to the two experimental factors of hydrogel treatment and time, as described in Section 4.8.

### 4.6. Histological and Immunohistochemical Analysis

At predetermined time points (days 3, 7, and 14) after treatment, five mice from each group were randomly selected and euthanized. The wound tissues together with the surrounding normal skin tissues were carefully collected for histological and immunohistochemical evaluation. The harvested tissue samples were immediately fixed in 4% paraformaldehyde for 24 h, followed by dehydration through a graded ethanol series, paraffin embedding, and sectioning into 5-μm-thick slices [59].

#### 4.6.1. Hematoxylin and Eosin (H&E) Staining

Paraffin-embedded tissue sections were deparaffinized with xylene, rehydrated through graded ethanol solutions, and stained with hematoxylin and eosin (H&E) according to standard protocols. After staining, sections were dehydrated, mounted, and observed using a light microscope. The morphological characteristics of regenerated wound tissues were evaluated, including inflammatory cell infiltration, granulation tissue formation, epidermal regeneration, and overall tissue structural restoration.

#### 4.6.2. Masson’s Trichrome Staining

Masson’s trichrome staining was performed to evaluate collagen deposition and extracellular matrix remodeling during wound healing. Briefly, paraffin sections were stained according to the manufacturer’s instructions. Collagen fibers were visualized as blue staining, while cellular components were stained in red. The distribution, density, and organization of collagen fibers within the wound area were observed by light microscopy.

#### 4.6.3. Immunohistochemical Staining

Immunohistochemical staining was performed to evaluate inflammatory responses and collagen formation in regenerated wound tissues. Paraffin sections were deparaffinized, rehydrated, and subjected to antigen retrieval using citrate buffer. After blocking endogenous peroxidase activity and nonspecific binding, sections were incubated overnight at 4 °C with primary antibodies against collagen I, IL-1β, and TNF-α. After washing, sections were incubated with corresponding secondary antibodies and developed using a chromogenic substrate. The stained sections were observed using a microscope, and the expression levels of target proteins were quantified by measuring the positive staining area using ImageJ software. For statistical analysis, each mouse was considered one independent biological replicate, and the quantitative immunohistochemical value obtained from the corresponding wound tissue was treated as one independent observation (*n* = 5 biological replicates per treatment group per time point).

### 4.7. Serum Inflammatory Cytokine Analysis

Blood samples were collected on days 3, 7, and 14 after treatment to evaluate systemic inflammatory responses during wound healing. Briefly, mice were anesthetized, and blood samples were collected from the orbital venous plexus under sterile conditions. The collected whole blood was transferred into non-anticoagulant centrifuge tubes and allowed to clot at room temperature. Subsequently, samples were centrifuged at 3000 rpm for 10 min at 4 °C to separate the serum. The obtained serum samples were carefully collected, aliquoted, and stored at −80 °C until further analysis. The concentrations of inflammatory cytokines, including IFN-γ, IL-1β, and TNF-α, were determined using enzyme-linked immunosorbent assay (ELISA) kits according to the manufacturer’s instructions. Briefly, serum samples and corresponding standards were added into designated wells and incubated with HRP-conjugated detection antibodies. After washing, substrate solutions were added for color development, and the reaction was terminated using stop solution. The absorbance values were measured at 450 nm using a microplate reader. The concentrations of target cytokines were calculated based on the corresponding standard curves. Each serum sample was obtained from an individual mouse and was treated as one independent biological replicate for statistical analysis (*n* = 5 biological replicates per treatment group per time point).

### 4.8. Statistical Analysis

All quantitative data are presented as mean ± standard deviation (SD). Data normality was assessed using the Shapiro–Wilk test. Comparisons among multiple groups with a single experimental factor were performed using one-way analysis of variance (ANOVA) followed by Tukey’s multiple-comparisons test. For datasets involving treatment and time as two independent factors, two-way ANOVA was performed, followed by Dunnett’s multiple-comparisons test. Statistical analyses were performed using GraphPad Prism 10.1.2 (GraphPad Software, Boston, MA, USA). A *p* value < 0.05 was considered statistically significant.

## 5. Conclusions

In this study, we extended the functional and in vivo evaluation of the previously developed CMC-HA-RGD hydrogel. In vitro agar-diffusion assays showed that CMC-HA-RGD exhibited the largest inhibition zones among the tested formulations, with inhibition-zone areas of approximately 2.0 cm^2^ against *E. coli* and 6.0 cm^2^ against *S. aureus*. In the mouse tail-transection model, the RGD-containing formulations showed reduced visible bleeding during the observation period, indicating favorable hemostatic behavior. In the full-thickness skin wound model, CMC-HA-RGD accelerated wound closure, reaching 95.56% closure on day 14. Histological analysis further showed a more complete epidermal–dermal structure, more abundant skin appendage-like structures, and denser collagen deposition in the CMC-HA-RGD group. Consistently, immunohistochemical analysis showed that, on day 14, CMC-HA-RGD reduced IL-1β- and TNF-α-positive areas to 10.16% and 10.40%, respectively, while increasing collagen I-positive area to 72.58% (*p* = 0.0042; *p* = 0.0034; *p* = 0.0005). Serum ELISA results further showed that IFN-γ, IL-1β, and TNF-α levels decreased to 155.83, 14.61, and 86.54 pg/mL, respectively, on day 14 (*p* = 0.0001; *p* = 0.0004; *p* = 0.0016). Taken together, the combined incorporation of HA and RGD improved the overall functional performance of the CMC-based hydrogel among the tested formulations, as reflected by its in vitro antibacterial activity, favorable hemostatic behavior, enhanced wound closure, lower levels of the examined inflammatory markers, and increased collagen deposition. These findings support the potential of CMC-HA-RGD as a multifunctional material for skin wound repair.

## Figures and Tables

**Table 1:** Physical and Chemical Properties of CMC, HA, and RGD.

Materials	Carboxymethyl Chitosan	Hyaluronic Acid	RGD Peptide
Molecular formula	[C_6_H_11_NO_4_(C_2_H_2_O_2_)_x_]_n_	(C_14_H_21_NO_11_)_n_	C_12_H_22_N_6_O_6_
Average polymer molecular weight	Not specified by supplier	1.0–1.3 MDa	Not applicable
Molecular weight	Not applicable	Not applicable	346.36 g/mol
Purity/specification	Degree of carboxymethylation ≥ 80%; viscosity 200–300 cps	Purity ≥ 97%; medium–high-molecular-weight grade	99.60%
Characteristic FTIR bands	~3420, 2920, 1600–1610, 1410, 1070–1080 cm^−1^	~3400, 2930, 1610, 1410, 1040 cm^−1^	~1650, 1540–1550, ~1300 cm^−1^
Thermal behavior	Main degradation ~200–300 °C	Main degradation ~195–300 °C	Melting point: 153–155 °C

## Data Availability

The raw data supporting the conclusions of this article will be made available by the corresponding authors on request.
